# Gender Invariance of the *Gambling Behavior Scale for Adolescents* (GBS-A): An Analysis of Differential Item Functioning Using Item Response Theory

**DOI:** 10.3389/fpsyg.2017.00940

**Published:** 2017-06-06

**Authors:** Maria Anna Donati, Francesca Chiesi, Viola A. Izzo, Caterina Primi

**Affiliations:** Dipartimento di Neuroscienze, Psicologia, Area del Farmaco e Salute del Bambino (NEUROFARBA), University of FlorenceFlorence, Italy

**Keywords:** gambling disorder, adolescents, gender invariance, differential item functioning, item response theory

## Abstract

As there is a lack of evidence attesting the equivalent item functioning across genders for the most employed instruments used to measure pathological gambling in adolescence, the present study was aimed to test the gender invariance of the *Gambling Behavior Scale for Adolescents* (GBS-A), a new measurement tool to assess the severity of Gambling Disorder (GD) in adolescents. The equivalence of the items across genders was assessed by analyzing Differential Item Functioning within an Item Response Theory framework. The GBS-A was administered to 1,723 adolescents, and the graded response model was employed. The results attested the measurement equivalence of the GBS-A when administered to male and female adolescent gamblers. Overall, findings provided evidence that the GBS-A is an effective measurement tool of the severity of GD in male and female adolescents and that the scale was unbiased and able to relieve truly gender differences. As such, the GBS-A can be profitably used in educational interventions and clinical treatments with young people.

## Introduction

International studies found gender differences in gambling problem severity among adolescents, indicating that boys are more likely than girls to report gambling problems (see Splevins et al., 2010; Calado et al., 2017, for reviews). Gender differences have been evidenced with different-aged samples belonging to various cultural contexts and by using different measurement tools (e.g., Delfabbro et al., 2009; Molde et al., 2009; Donati et al., 2013; Gupta et al., 2013). These instruments include the most employed scales used internationally, such as the *South Oaks Gambling Screen-Revised for Adolescents* (SOGS-RA; Winters et al., 1993), the *Diagnostic and Statistical Manual—Fourth Edition* [DSM-IV; American Psychiatric Association (APA), 1994], *Adapted for Juveniles* (DSM-IV-J; Fisher, 1992) and its revised version, the *Multiple-Response Format for Juveniles* (DSM-IV-MR-J; Fisher, 2000), and the *Massachusetts Adolescent Gambling Screen* (MAGS; Shaffer et al., 1994). Across the studies, gender differences have been detected by comparing the prevalence rates for each gambling problem severity category. In detail, classifying adolescent gamblers in non-problem, at-risk, and problem gamblers, boys have been found to be more likely to show at-risk and problem gambling behavior than girls, which have been more likely to be non-problem gamblers.

As the prevalence rates of gambling problem severity categories basically derive from the respondents' endorsement of test items, the question that arises is whether the employed instruments are gender-invariant, i.e., if a randomly selected girl with a specific level of gambling-related problems and a randomly selected boy with the same level of gambling-related problems have the same chance to endorse the items of a scale measuring problem gambling. Indeed, if this is not the case, a test is not metrically invariant, i.e., it does not measure the same construct in the same way in different groups because the responses to the items (or part of them) are related to group membership and not to the measured construct. As a consequence, the comparison of test scores between different groups of individuals has to be not considered valid (Waiyavutti et al., 2011).

Referring to the above mentioned measurement tools employed in adolescent gambling research, there is a lack of studies investigating their measurement invariance. Only Molde et al. (2009), using Item Response Theory (IRT), tested the Differential Item Functioning (DIF) across genders of the MAGS. The analysis of DIF is central to the investigation of the measurement equivalence of a scale at the item level because it allows to ascertain whether the response to an item is related to group membership and not to the measured construct (i.e., if a measure is biased because people, which belong to *different* group but hold the *same* characteristics with respect to the measured construct, answer differently). Molde et al. (2009) showed that all the items of the scale functioned differently in male and female respondents. As such, the gender differences observed employing this scale might be misleading because it is not possible to ascertain if they reflect actual differences in problem gambling among male and female adolescents or if they reflect differences related to group membership.

Following this premise, testing gender measurement invariance of the tools employed to assess pathological gambling in adolescents should be considered a prerequisite to investigate gender differences. Thus, the aim of the present work was to investigate whether the *Gambling Behavior Scale for Adolescents* (GBS-A; Primi et al., 2015) was gender-invariant in measuring pathological gambling severity in male and female gamblers. Specifically, to offer evidence that the GBS-A was gender invariant, we aimed to test its equivalence across genders by exploring DIF within the IRT framework, which allows us to assess whether the test items measure problem gambling *fairly* in boys and girls.

In respect of the above mentioned scales, which were developed before the fifth *Diagnostic and Statistical Manual of Mental Disorders* [DSM-5; American Psychiatric Association (APA), 2013], the GBS-A is a scale for adolescents that measures gambling habits and Gambling Disorder (GD), as conceptualized in the last edition of the DSM, which includes the specifications that different and progressive levels (mild, moderate, severe) of GD severity can occur and that GD may apply also to adolescents and young people. Additionally, the scale was chosen because it was developed taking into account the largely shared indication that different aspects of problem gambling are not equivalent indicators of pathology (e.g., Shaffer et al., 1994; Wiebe et al., 2000; Derevensky and Gupta, 2004; Colasante et al., 2014; Edgren et al., 2016). In particular, to fit with this indication, the scale was developed applying IRT. Indeed, inside the IRT framework, one of the item characteristics is its location, which can be conceptualized as the “severity” of the symptom described by the item. Thus, applying a IRT-based scoring procedure, the GBS-A allows to measure GD taking into account the relative weight (i.e., the severity) of each symptom described by the items of the scale.

Finally, given the large consensus about the fact that boys hold higher levels of GD severity than girls (see Splevins et al., 2010; Calado et al., 2017, for reviews), we aimed to test if the GBS-A was able to confirm this difference in GD between male and female adolescent gamblers. In detail, we wanted to explore the gender differences and similarities in the GBS-A items endorsement, in the total score, and in the derived classification into non-problem gamblers, at-risk gamblers, and disordered gamblers.

## Methods

### Participants

Participants were 1,723 (56% males) 11- to 23-year-old students attending middle and secondary schools in suburban and urban school districts in Italy with a mean age of 15.64 years (*SD* = 1.79). The data collection took place in agreement with the schools (the research project was approved by the schools' local ethical committee) and following the requirements of privacy and informed consent requested by Italian law (Legislative Decree DL-196/2003). In detail, written informed assent was provided by students and written informed consent was provided by the parents if the student was a minor. Regarding the ethical standards for research, the study referred to the last version of the Declaration of Helsinki (World Medical Association, 2013).

### Measures and procedure

The GBS-A (Primi et al., 2015) is composed of two sections. The first one consists of unscored items investigating gambling behavior. Specifically, these items assess the frequency (never, sometimes in the year, sometimes in the month, sometimes in the week, daily) of participation during the last year in ten gambling activities (card games, bets on games of personal skill, bets on sports games, bets on horse races, bingo, slot machines, scratch cards, lotteries, online games, and private bets with friends), gambling versatility, the gambling partners (alone, with friends, with boyfriend/girlfriend, with someone of the family), relative gambling frequency with them (never, sometimes, often), and the amount of money spent on gambling.

The second section is composed of nine items, each one developed in order to relieve one of the nine DSM-5 diagnostic criteria of GD among adolescents. An example of item is “*Have you spent in gambling money intended for other purposes?*” All items have a three-response format, i.e., 0 = never, 1 = sometimes, 2 = often. This scale was proved to be unidimensional and the Test Information Function (TIF), which is used to evaluate the precision of the test at different levels of the measured construct, showed that the instrument was highly informative for mid- to high-levels of severity of GD. Validity measures were provided showing significant relationships with gambling frequency, problem gambling (as measured by the SOGS-RA; Italian version: Colasante et al., 2014), and a large array of risk factors for gambling problems, such as gambling-related cognitive distortions, sensation seeking, superstitious thinking, pressure to conform to peers, and social independence from peers.

Based on the responses to this section, for each respondent is possible to derive a IRT-based score, which basically consists in a sum of the frequency by which each of the items endorsed have been experienced, weighted on the specific severity and discrimination parameters characterizing these items. Following this IRT-based scoring procedure, respondents can be classified into non-problem gamblers, at-risk gamblers, and disordered gamblers (Primi et al., 2015).

The GBS-A was administered within the classrooms and during school time by professionally trained researchers. The students were provided with a brief introduction to the study, and with some instructions. Each participant worked individually. Answers were collected in a paper-and-pencil format, and data collection was completed in about 20 min.

### Data analysis

Preliminarily, we measured gambling frequency, gambling versatility, gambling partners, and the amount of money spent on gambling by gender. Then, considering the second section, analyses of DIF across genders were performed by applying the IRT Likelihood Ratio test approach implemented in IRTPRO (Cai et al., 2011) and, according to the response format, Samejima's (1969) graded response model (GRM), one of the most used models for graded polytomous data, was chosen.

Prior to conduct the DIF gender analyses, we looked at the assumptions of the unidimensionality and the item fit under the GRM in each gender group. The unidimensionality of the scale was evaluated by the presence of local dependence (LD) and a χ^2^ LD statistic was used. Values equal to 10 or greater indicate an excess in covariation among item responses that is not explained by the unidimensional model. Then, the item fit under the GRM was tested for each item by computing the *S*-χ^2^ statistics (Orlando and Thissen, 2000). Significant *S*-χ^2^ statistics indicate that the item did not fit under the model (Hambleton et al., 1991; Hambleton and Han, 2005). Given that using larger samples results in a greater likelihood of significant chi-square differences, the critical value of 0.01 rather than the usual critical value of 0.05 was employed (Stone and Zhang, 2003).

The DIF detection procedure is based on a nested model comparison approach. First, a more parsimonious model is tested with all parameters (β and α) constrained to be equal across groups for a studied item against an augmented model. Here, one or more parameters of studied item are freed to be estimated distinctly for the two groups (a focal group and a reference group). This procedure involves comparing differences in log-likelihoods (distributed as chi-square) associated with nested models. Since multiple tests were performed, the level of significance of 0.05 was adjusted by Bonferroni correction to 0.003 (0.05/16).

Finally, gender differences were investigated by looking at the item distribution by gender and by comparing across genders the total score of the IRT-based GBS-A score and the distribution of non-problem, at-risk, and disordered gamblers.

## Results

Results showed that 30% of the participants had never gambled. We performed the analyses on adolescent gamblers, i.e., the 1,201 respondents (59% males, mean age = 15.66, *SD* = 1.71) who affirmed having gambled at least once during the last year. Concerning missing data treatment, when missing values did exceed 10% of total answers, cases were excluded. When missing values did not exceed 10% of total answers, the *Expectation-Maximization* (EM) estimation method (Bock and Aitkin, 1981) was used to replace missing data. Only 1.2% (*n* = 14) of the respondents were excluded, thus IRT analyses were performed on a sample of 1,187 cases (59% males, mean age: 15.66, *SD* = 1.71).

Data showed that the groups of male and female gamblers were homogeneous in terms of age (Male adolescents: mean age = 15.68, *SD* = 1.67; Female adolescents: mean age = 15.65, *SD* = 1.77, *p* = 0.766), and level of education (Male adolescents: 11% middle school, 89% high school; Female adolescents: 14% middle school, 86% high school, *p* = 0.139).

Concerning descriptive data relative to the GBS-A first section, results showed that the most engaged gambling activities among boys were bets on sport games, scratch cards, and bingo, while girls preferred to gamble on bingo, followed by scratch cards and card games. Furthermore, while boys were used to gamble with friends, girls preferred someone of the family (Table 1). Additionally, male (*M* = 3.24, *SD* = 2.17) and female adolescents (*M* = 3.06, *SD* = 1.93) gambled on a similar number of activities [*t*_(1, 185)_ = 1.46, *p* = *0*.145]. Finally, boys (*M* = 29.67 €, *SD* = 48.43) spent higher amount of money on gambling than girls (*M* = 18.75 €, *SD* = 41.47) [*t*_(755)_ = 4.40, *p* < 0.001, Cohen's *d* = 0.24].

Table 1Gambling frequency for each activity and for gambling partners by gender.

**Table:** 

**Gambling activities**	**Never**		**Sometimes in the year**		**Sometimes in the month**		**Sometimes in the week**		**Daily**		**Total gamblers**	
	**Males (%)**	**Females (%)**	**Males (%)**	**Females (%)**	**Males (%)**	**Females (%)**	**Males (%)**	**Females (%)**	**Males (%)**	**Females (%)**	**Males (%)**	**Females (%)**
Card games	53.5	57.8	26.9	28.3	10.3	9.4	7.3	3.3	2.0	1.2	46.5	42.2
Bets on games of personal skill	71.0	68.6	16.5	20.7	7.9	8.2	3.3	2.0	1.4	0.4	29.0	31.4
Bets on sport games	44.2	74.0	17.3	13.9	14.6	5.1	19.0	4.7	4.9	2.3	55.8	26.0
Bets on horse races	91.3	90.0	5.2	6.8	2.1	1.4	1.1	1.6	0.3	0.2	8.7	10.0
Bingo	52.8	38.7	38.9	50.2	5.7	7.8	1.7	2.7	0.9	0.6	47.2	61.3
Slot machines	89.1	94.1	6.3	4.9	2.3	1.0	1.6	–	0.7	–	10.9	5.9
Scratch cards	46.9	41.6	34.8	43.6	13.9	11.1	3.1	3.1	1.3	0.6	53.1	58.4
Lotteries	75.3	74.8	17.5	19.3	4.3	3.3	2.3	2.0	0.7	0.6	24.7	25.2
Online games	81.7	84.8	7.2	6.8	3.1	3.3	4.0	3.1	4.0	2.0	18.3	15.2
Private bets with friends	75.8	86.4	11.8	10.4	7.3	1.6	3.4	1.6	1.7	–	24.2	13.6

**Table:** 

**Gambling partners**	**Never**		**Sometimes**		**Often**		**Total gamblers**	
	**Males (%)**	**Females (%)**	**Males (%)**	**Females (%)**	**Males (%)**	**Females (%)**	**Males (%)**	**Females (%)**
Alone	71.4	87.2	20.5	9.3	8.0	3.5	28.6	12.8
With friends	29.3	47.9	36.4	37.5	34.3	14.5	70.7	52.1
With boyfriend/girlfriend	81.1	76.0	13.3	17.2	5.7	6.8	18.9	24.0
With someone of the family	37.1	21.0	36.9	45.3	26.0	33.6	62.9	79.0

*The percentages are in relation to the gender variable*.

### Gender measurement invariance

The results confirmed that a single factor model adequately represented the structure of the scale for each group, as none of the LD statistics were >10. The Samejima's (1969) GRM model was tested. Both in male and female gamblers, each item had a non-significant (*p* > 0.01) *S*-χ^2^ value (Table 2), indicating that all items fit under the GRM model.

**Table 2:** Fit statistics, parameters for each item of the GBS-A for gender groups, and DIF analysis of discrimination and severity parameters across genders.

		**Males**					**Females**					**aDIF**			**bDIF**		
**Item**	**DSM-5 criterion**	**S-χ^2^ (*df*)**	***p***	**a (SE)**	**b_1_ (SE)**	**b_2_ (SE)**	**S-χ^2^ (df)**	***p***	**a (SE)**	**b_1_ (SE)**	**b_2_ (SE)**	**χ^2^**	***df***	***p***	**χ^2^**	***df***	***p***
1	Tolerance	11.15 (12)	0.517	3.56 (0.54)	1.52 (0.13)	2.71 (0.27)	3.89 (7)	0.793	3.27 (0.82)	1.92 (0.19)	3.07 (0.41)	0.1	1	0.707	0.3	2	0.869
2	Withdrawal	16.19 (17)	0.512	3.00 (0.43)	1.37 (0.12)	2.17 (0.20)	19.71 (10)	0.032	2.82 (1.22)	1.91 (0.25)	2.78 (0.48)	0.2	1	0.622	1.8	2	0.414
3	Loss of control	20.30 (16)	0.207	3.42 (0.60)	1.56 (0.13)	2.27 (0.21)	7.69 (8)	0.465	2.20 (0.42)	2.15 (0.24)	3.33 (0.51)	1.0	1	0.311	0.7	2	0.708
4	Preoccupation	36.07 (19)	0.012	1.98 (0.25)	1.16 (0.11)	2.04 (0.20)	10.62 (13)	0.644	1.65 (0.29)	1.77 (0.22)	2.61 (0.34)	0.0	1	0.979	3.4	2	0.186
5	Escape	15.68 (16)	0.477	2.34 (0.36)	1.59 (0.15)	2.76 (0.30)	6.04 (5)	0.304	2.40 (0.41)	1.63 (0.16)	3.73 (0.63)	0.8	1	0.386	5.2	2	0.074
6	Chasing	15.40 (16)	0.497	1.86 (0.22)	0.75 (0.09)	2.53 (0.24)	15.90 (10)	0.102	1.35 (0.22)	0.96 (0.14)	3.40 (0.48)	0.5	1	0.467	2.0	2	0.368
7	Lying	15.36 (18)	0.638	2.58 (0.35)	1.35 (0.12)	2.31 (0.22)	6.30 (8)	0.614	2.29 (0.47)	2.04 (0.22)	3.45 (0.52)	0.1	1	0.809	5.9	2	0.053
8	Risked/lost relationships, opportunities	16.01 (14)	0.312	3.27 (0.76)	1.58 (0.15)	2.46 (0.28)	14.74 (8)	0.064	3.34 (0.44)	1.73 (0.13)	2.63 (0.24)	0.1	1	0.719	4.3	2	0.117
9	Bail-out	25.12 (21)	0.241	1.78 (0.23)	1.42 (0.15)	2.74 (0.30)	13.68 (11)	0.251	1.85 (0.32)	1.71 (0.21)	3.22 (0.45)	0.9	1	0.339	0.7	2	0.714

*Parameters were computed under the GRM model (a, discrimination; b, severity). df, degrees of freedom; SE, standard error. Due to the large sample size α was fixed at 0.01*.

The gender DIF analyses (in which the male group was the reference group) showed from the first step that no items showed DIF (item DIF statistics ranged from 0.0 to 5.9, with associated *p*-values ranging from 0.979 to 0.053; Table 2). Thus, the GBS-A can be considered invariant across genders. Concerning the parameters, the GRM is a two-parameter model referring to the item severity and discrimination. Specifically, given the 3-point response format of the scale, two threshold parameters (β_*i*_)—equal to the number of response options minus 1—are derived indicating the trait level where there is a 0.5 probability of endorsing the relevant response option or higher response options. Values can be interpreted as the “severity” of the symptom described by the item, i.e., higher the level of the trait on which the threshold are located, higher the severity of the item. Since in both groups the β_1_ values were around 1 *SD* above the mean trait level (fixed at 0.00, *SD* = 1.00, by default) and β_2_ at around 2 *SDs* above the mean trait level, all items can be considered very severe. The discrimination parameter (*a*) indicates the ability of an item to discriminate among people holding different levels of the underlying trait. According to Baker and Kim (2004), values 0.01–0.24 are very low, 0.25–0.64 are low, 0.65–1.34 are moderate, 1.35–1.69 are high, and more than 1.7 are very high. The item *a* values (between 1.78 ± 0.23 and 3.56 ± 0.54 among male gamblers and between 1.34 ± 0.22 and 3.34 ± 0.44 among female) indicated a high or very high discriminative ability.

### Gender differences

The descriptive statistics for each item were calculated for boys and girls (Table 3). Overall, results showed slightly higher percentages of “never” responses in girls. As such, boys showed higher endorsement of the “sometimes” and “often” options. However, the distributions for tolerance, escape, chasing and risked/lost relationships and opportunities items/criterions were quite similar.

**Table 3:** Percentages of item endorsement for each response option of the GBS-A across genders.

		**Males**			**Females**		
**Item**	**DSM-5 criterion**	**Never**	**Sometimes**	**Often**	**Never**	**Sometimes**	**Often**
1	Tolerance	91.4	7.9	0.7	95.4	4.3	0.4
2	Withdrawal	88.0	8.9	3.1	94.7	4.3	1.0
3	Loss of control	91.8	6.0	2.1	94.9	4.5	0.6
4	Preoccupation	80.1	13.3	6.6	88.9	7.2	3.9
5	Escape	89.6	9.0	1.4	90.6	9.2	0.2
6	Chasing	70.7	25.9	3.4	73.0	24.8	2.3
7	Lying	86.6	10.6	2.9	94.5	5.1	0.4
8	Risked/lost relationships, opportunities	91.7	6.9	1.4	93.6	5.3	1.0
9	Bail-out	84.7	12.6	2.7	89.3	9.4	1.2

Considering the total score of the GBS-A, results showed that the IRT-based score values ranged from 0 to 18.90 among boys and from 0 to 16.90 among girls. A significant difference was found between male (*M* = 1.73, *SD* = 3.01) and female adolescents (*M* = 1.12. *SD* = 2.18), who showed significantly [*t*_(1185)_ = 3.86, *p* < 0.001, Cohen's *d* = 0.23] lower values.

According to the criterion described by Primi et al. (2015), adolescents were classified into non-problem gamblers, at-risk gamblers, and disordered gamblers. There was a significant difference in the percentage distribution of the three categories of gamblers between boys and girls [χ^2^(2, *N* = 1,187) = 15.21, *p* < 0.001, *V Cramer* = 0.113]. More girls than boys were *non-problem gamblers* (90 and 81%, respectively), while boys showed higher rates of *at-risk gambling* (12%) and *disordered gambling* (7%) than girls did (7 and 3%, respectively).

## Discussion

Gender differences in adolescent gambling behavior have been widely documented and discussed (see Merkouris et al., 2016, for a recent systematic review). Consistent with past research (e.g., Donati et al., 2013), this study confirmed gender-specific preferences in engagement on gambling. Indeed, boys preferred to gamble on bets on sport games and girls on bingo, male adolescents gambled mostly with friends while female adolescents with someone of the family. Furthermore, the fact that boys spent more money on gambling than girls is in line with past studies (e.g., Felsher et al., 2004). Given these differences in gambling habits, it is important to deeply investigate gender differences related to GD symptoms.

Indeed, as research has found substantial gender differences in the prevalence of pathological gambling (see Splevins et al., 2010; Calado et al., 2017, for reviews), it is important to analyze whether the scales used are invariant across male and female adolescent gamblers, following the suggestion that “*fair measurement requires that test scores have the same meaning across all relevant examinee groups*” (Reise and Waller, 2009, p. 37). Nevertheless, to the best of our knowledge, with one exception, the most internationally employed instruments have not proved to be invariant across genders. As a consequence, in comparing test scores between male and female adolescents, we cannot exclude that the instruments fail to measure the construct in the same way in boys and girls. By applying IRT analyses, this study shows that the GBS-A (Primi et al., 2015), a new instrument recently developed for measuring the severity of GD among youth, is invariant across genders, i.e., we attested the measurement equivalence of the scale when administered to male and female adolescents. This ensures that the GBS-A can be used to compare boys' and girls' measure of pathological gambling and group differences can be interpreted in terms of differences in the underlying construct.

This finding appears to be important for adolescent gambling research because the other tool for which the measurement invariance was tested, i.e., the MAGS (Molde et al., 2009), showed a differential functioning across genders. Additionally, results from research with adults have evidenced gender-related biases concerning the DSM diagnostic criteria for pathological gambling. In detail, using Rasch modeling techniques, Strong and Kahler (2007) found that, given the same latent trait, women were more likely to endorse gambling to escape. Through Multiple-Indicator Multiple-Cause (MIMIC) modeling, Sacco et al. (2011) confirmed the DIF across genders for escape criterion and also found that men were more likely to endorse preoccupation.

Along with GBS-A gender invariance, some other important results have been provided by this study. First, the scale has been found to be unidimensional both in male and female adolescent gamblers, in line with the definition of GD in the DSM-5. Second, IRT attested that item properties (i.e., severity and discrimination) in male and female adolescents were consistent with the aim of measuring GD efficiently. With regard to severity, both in boys and girls, all the items resulted to be located along the range of the continuum that the scale was aimed to measure accurately, i.e., from at-risk to disordered gambling behavior. This indicated that the items adequately covered the range of the latent trait. Concerning discrimination, the parameter estimates indicated that the items of the GBS-A were able to distinguish between the different levels of the trait in boys and girls.

Finally, the GBS-A resulted to relieve the expected gender difference in GD (e.g., Delfabbro et al., 2009; Molde et al., 2009; Donati et al., 2013; Gupta et al., 2013). Specifically, the gender-specific endorsement for each item option revealed higher affirmative endorsement rates in boys. As such, male adolescents resulted to have higher levels of GD compared with female adolescents and a higher prevalence of both at-risk gamblers and disordered gamblers was found among boys rather than girls. This finding confirms and strengths previous results on gambling gender difference in adolescence given the gender measurement equivalence of the scale employed to assess problem gambling.

In terms of practical implications, the GBS-A can therefore be used both in research and practice. As for research, it appears to be as a useful instrument to be used to identify male and female adolescent gamblers characterized by pathological levels of gambling and to analyze gender differences and similarities in the predictors of disordered gambling among adolescents. In this regard, relatively few studies have until now analyzed gender specificity of the predictors of pathological gambling in adolescents (e.g., Chalmers and Willoughby, 2006; Jackson et al., 2008; Donati et al., 2013); thus, it is not clear yet whether the predictors of gambling involvement are similar for male and female adolescents. By applying the GBS-A, future studies should be conducted in order to extend knowledge about this issue.

For practitioners, the GBS-A can be profitably used in educational interventions and clinical treatments. From an educational point of view, it could be used as a measurement tool to evaluate the effectiveness of preventive interventions aimed to reduce gambling behavior among male and female adolescents. Specifically, the scale can be applied to have a reliable and valid measurement of the situation of participants' gambling behavior at the baseline, after the intervention, and at the follow-up. Specifically, as reviewed by Edgren et al. (2016), among the most employed instruments to measure the severity of gambling problems in youth, only the SOGS-RA has been used to verify the effectiveness of preventive interventions in decreasing the severity of gambling problems (Hansen and Rossow, 2010; Donati et al., 2014). As regards its clinical application, the GBS-A could be used with at-risk adolescents in order to assess the severity of GD. Indeed, several studies have shown that substance abuse, excessive use of alcoholics and driving under the influence of alcohol are associated with pathological gambling behavior among adolescents (e.g., Gupta et al., 2004; Splevins et al., 2010; Gori et al., 2014). For these reasons, when juveniles with these problems are detected, it may be done an assessment of gambling behavior by applying the GBS-A.

The present study offers several notable strengths, e.g., the large sample size and the application of IRT models to analyze DIF of the GBS-A. Nevertheless, some limitations have to be acknowledged. Specifically, as we recruited our sample in schools, participants were all adolescents attending middle and high school, whereas students who dropped out of school or working adolescents were not included. Furthermore, whereas the characteristics of the gambling phenomenon of the present study measured with the GBS-A are in line with the international literature, this study has been conducted with Italian adolescents, and some limitations regarding external validity might be related to the specificity of the sample. To overcome these limitations, measurement equivalence across country should be verified in future studies by checking the invariance of the scale across national contexts. It should be also interesting to test the psychometric properties of the scale in different populations, such as clinical sample of adolescents.

In sum, overall our results provide evidence that the GBS-A is psychometrically appropriate to be used with boys and girls. Thus, it can be used by researchers and practitioners dealing with the issue of understanding, prevention and treatment of problem gambling among adolescents.

## Ethics statement

This study was carried out in accordance with the recommendations of APA and with written informed consent from all subjects. All subjects gave written informed consent in accordance with the Declaration of Helsinki. The protocol was approved by the ethical committees of each involved school.

## Author contributions

MD developed the research project and conducted the test administration in the school classrooms. She developed the analyses and wrote the paper. FC collaborated in the data analyses and the paper writing. VI collaborated in the test administration and data enter/analyses, while CP supervised the entire work and gave her contribution in the finding discussion.

### Conflict of interest statement

The authors declare that the research was conducted in the absence of any commercial or financial relationships that could be construed as a potential conflict of interest.
